# Supplementary material for: Development of a Sport Food Exchange List for Dietetic Practice in Sport Nutrition
Source: Nutrients. 2020 Aug 11;12(8):2403. doi: 10.3390/nu12082403 (PMC7468869; doi:10.3390/nu12082403)
Supplement: Supplementary file 1 [file nutrients-12-02403-s001.pdf]

Table S1. Sports Foods Exchange Lists.

SPORTS DRINKS GROUP	EXCHANGE VALUE			138	0,04	33	0,01	1	-0,17	0,00	-0,21	
**Produt name**	**Brand name**	**Net weight (g or ml)**	**Serving dosage**	**Energy (Kcal)**	**Z value**	**Carbohydrate (g)**	**Z value**	**Protein (g)**	**Z value**	**Fat (g)**	**Z value**	**Sodium (mg)**
				<250 mg sodium								
Nd3	Infisport	30	2 serving	115,00	-1,15	27,50	-1,47	1,25	0,13	0,00	0,00	0,00
Amylopectin	Infisport	40	3 serving	144,00	0,30	36,00	0,80	0,00	-0,52	0,00	0,00	0,00
Bebida isotónica – limón	Enervit	30	2 serving	109,80	-1,41	27,50	-1,48	0,00	-0,52	0,00	0,00	144,00
Nutrixxion energía bebida endurance xx force	Nutrixxion	35	2 serving	123,64	-0,72	30,20	-0,75	0,71	-0,15	0,00	0,00	245,20
Nutrixxion energía bebida endurance orange	Nutrixxion	35	2 serving	129,41	-0,43	32,10	-0,23	0,21	-0,41	0,00	0,00	245,20
Nutrixxion energía bebida endurance lemon	Nutrixxion	35	2 serving	129,41	-0,43	32,10	-0,23	0,21	-0,41	0,00	0,00	245,20
Nutrixxion energía bebida endurance redfruit	Nutrixxion	35	2 serving	129,11	-0,45	32,10	-0,25	0,22	-0,41	0,00	0,00	245,20
Iso drink refresher 700g citrus	Nutrixxion	35	2 serving	128,88	-0,46	32,20	-0,21	0,00	-0,52	0,00	0,00	245,20
Nutrixxion iso bebida refresher grapefruit	Nutrixxion	35	2 serving	126,56	-0,57	31,60	-0,36	0,00	-0,52	0,00	0,00	244,00
Isotonic drink - sabor limon	226ers	40	3 serving	136,00	-0,10	34,00	0,27	0,00	-0,52	0,00	0,00	196,00
Isotonic drink - sabor frutos rojos	226ers	40	3 serving	136,00	-0,10	34,00	0,27	0,00	-0,52	0,00	0,00	196,00
Isotonic drink - sabor mango	226ers	40	3 serving	136,00	-0,10	34,00	0,27	0,00	-0,52	0,00	0,00	196,00
Isotonic drink - sabor cola	226ers	40	3 serving	136,00	-0,10	34,00	0,27	0,00	-0,52	0,00	0,00	196,00
Energy drink - lemon	226ers	37,5	3 serving	132,00	-0,30	33,00	0,00	0,00	-0,52	0,00	0,00	24,00
Energy drink - mandarina	226ers	37,5	3 serving	132,00	-0,30	33,00	0,00	0,00	-0,52	0,00	0,00	24,00
Energy drink - furtos rojos	226ers	37,5	3 serving	135,00	-0,15	33,80	0,20	0,00	-0,52	0,00	0,00	24,00
Iso carbo organge	Victory endurance	40	2 servirg	133,20	-0,24	33,30	0,08	0,00	-0,52	0,00	0,00	162,70
Energy carbo charge chocolate	Keepgoing	55	3 serving	195,45	2,88	41,50	2,27	5,00	2,09	1,05	6,56	44,00
Longovit 360 mango-maracuyá	Nutrinovex	40	2 servirg	141,27	0,16	34,50	0,40	0,76	-0,13	0,03	0,16	0,00

				>250 mg sodium								
Isodrink & energy limón	Crown sport nutrition	48	3 serving	170,40	1,62	39,00	1,60	3,60	1,36	0,00	0,00	345,00
Isodrink & energy frutos rojos	Crown sport nutrition	48	3 serving	170,40	1,62	39,00	1,60	3,60	1,36	0,00	0,00	345,00
Aubisque	Geo	35	2 serving	128,00	-0,50	32,00	-0,27		-0,52		0,00	375,00
Nd4	Infisport	39	2 serving	132,00	-0,30	30,00	-0,80	3,00	1,05	0,00	0,00	500,00
Drink salts sabor limón	Infisport	40	2 sreving	144,00	0,30	36,00	0,80	0,00	-0,52	0,00	0,00	575,00
Drink salts sabor naranja	Infisport	40	2 sreving	140,00	0,10	35,00	0,53	0,00	-0,52	0,00	0,00	575,00
Hydrasport	Recuperat-ion	40	2 serving	128,00	-0,50	32,00	-0,27	0,00	-0,52	0,00	0,00	1036,00
Enervit g endurance sports con cafeína	Enervit	45	3 serving	154,80	0,84	38,70	1,52	0,00	-0,52	0,00	0,00	315,00
Sub9 energy drink - watermelon	226ers	37,5	3 serving	132,00	-0,30	28,50	-1,20	4,50	1,83	0,00	0,00	497,93
Sub9 energy drink - mango	226ers	37,5	3 serving	130,40	-0,38	28,10	-1,31	4,50	1,83	0,00	0,00	498,08
Iso energy orange	Victory endurance	40	2 servirg	136,00	-0,10	32,00	-0,27	2,00	0,52	0,00	0,00	272,00
Iso energy orange-mandarina	Victory endurance	40	2 servirg	136,00	-0,10	32,00	-0,27	2,00	0,52	0,00	0,00	272,00
Iso energy lemon	Victory endurance	40	2 servirg	141,20	0,16	33,30	0,08	2,00	0,52	0,00	0,00	272,00
Iso energy ice blue	Victory endurance	40	2 servirg	136,00	-0,10	32,00	-0,27	2,00	0,52	0,00	0,00	272,00
Nitro energy drink blood orange	Victory endurance	40	2 servirg	148,40	0,52	34,70	0,45	2,40	0,73	0,00	0,00	368,00
Bebida isotónica orange	Weider	40	2 servirg	149,20	0,56	37,30	1,15	0,00	-0,52	0,00	0,00	336,00
Isotonic	Etixx	35	2 servirg	124,80	-0,66	31,20	-0,48	0,00	-0,52	0,00	0,00	276,00
Isotonic orange	Etixx	35	2 servirg	121,60	-0,82	30,40	-0,69	0,00	-0,52	0,00	0,00	280,00
Isotonic orange-mango	Etixx	35	2 servirg	124,80	-0,66	31,20	-0,48	0,00	-0,52	0,00	0,00	276,00
Isotonic watermelon	Etixx	35	2 servirg	124,80	-0,66	31,20	-0,48	0,00	-0,52	0,00	0,00	276,00
Powerade® ice storm	Cocacola	500	1 serving	100,00	-1,91	25,00	-2,13	0,00	-0,52	0,00	0,00	260,00
Powerade® citrus charge	Cocacola	500	1 serving	104,00	-1,71	26,00	-1,87	0,00	-0,52	0,00	0,00	260,00

Powerade® blood orange	Cocacola	500	1 serving	100,00	-1,91	25,00	-2,13	0,00	-0,52	0,00	0,00	260,00
Triforza energy lemon	Keepgoing	50	3 serving	172,98	1,75	37,70	1,25	5,32	2,26	0,10	0,63	360,00
Triforza energy hawaiian tropical	Keepgoing	50	3 serving	171,69	1,69	37,40	1,17	5,32	2,26	0,09	0,56	356,00
Triforza energy green apple	Keepgoing	50	3 serving	173,28	1,77	37,73	1,26	5,32	2,26	0,12	0,75	360,00
Triforza energy mixed berries	Keepgoing	50	3 serving	170,13	1,61	37,00	1,07	5,33	2,27	0,09	0,56	356,00
			**Media**	**137,25**		**32,95**		**1,32**		**0,03**		
			**Deviation**	**19,94**		**3,75**		**1,91**		**0,16**		
			**Coefficient of Variation (CV)**	**14,53**		**11,38**		**144,96**		**481,04**		

**SPORTS GELS GROUP**	**EXCHANGE VALUE**			**101**	**-0,02**	**25,00**	**-0,03**	**0**	**-0,57**	**0**	**-0,40**	
**Produt name**	**Brand name**	**Net weight (g or ml)**	**Serving dosage**	**Energy (Kcal)**	**Z value**	**Carbohydrate (g)**	**Z value**	**Protein (g)**	**Z value**	**Fat (g)**	**Z value**	**Sodium (mg)**
				**< 100 mg sodium**								
Gel energy cítrico	Biofrutal sport	30	1 serving	92,40	-0,50	23,10	-0,45	0,00	0,00	0,00	0,00	0,00
Gel energy manzana	Biofrutal sport	30	1 serving	92,40	-0,50	23,10	-0,45	0,00	0,00	0,00	0,00	0,00
Gel energy sprint final	Biofrutal sport	30	1 serving	102,00	0,06	25,50	0,12	0,00	0,00	0,00	0,00	0,00
Gel energy sprint final + cafeína	Biofrutal sport	30	1 serving	98,40	-0,15	24,60	-0,09	0,00	0,00	0,00	0,00	0,00
Gluco gel	Glucoup! - gluco	50	2 serving	113,44	0,72	28,36	0,79	0,00	0,00	0,00	0,00	0,00
Gluco juice	Glucoup! - gluco	120	2 serving	121,60	1,19	30,40	1,27	0,00	0,00	0,00	0,00	0,00
Pump gel lemon	Victory endurance	42	1 serving	96,00	-0,29	24,00	-0,24	0,00	0,00	0,00	0,00	0,00
Energy gels	Etixx	38	1 serving	96,00	-0,29	24,00	-0,24	0,00	0,00	0,00	0,00	0,00
Ginseng and Guarana Energy Gel	ETIXX	50	1 serving	120,00	1,10	30,00	1,18	0,00	0,00	0,00	0,00	0,00
Enervit sport liquid gel – te verde	Enervit	60	1 serving	112,80	0,68	28,20	0,75	0,00	0,00	0,00	0,00	3,12
Enervit sport liquid gel – limón	Enervit	60	1 serving	112,80	0,68	28,20	0,75	0,00	0,00	0,00	0,00	3,12

Enervit sport liquid gel – naranja	Enervit	60	1 serving	112,80	0,68	28,20	0,75	0,00	0,00	0,00	0,00	3,12
Enervit sport liquid gel competition – cereza	Enervit	60	1 serving	112,80	0,68	28,20	0,75	0,00	0,00	0,00	0,00	3,12
Enervite sport liquid gel competition – cítricos	Enervit	60	1 serving	112,32	0,65	28,08	0,72	0,00	0,00	0,00	0,00	3,12
Energy boost gel orange	Victory endurance	42	1 serving	97,60	-0,20	24,40	-0,14	0,00	0,00	0,00	0,00	13,00
Hydro energy apple	Victory endurance	70	1 serving	96,00	-0,29	24,00	-0,24	0,00	0,00	0,00	0,00	14,00
Hydro energy caffeine red fruits	Victory endurance	70	1 serving	96,00	-0,29	24,00	-0,24	0,00	0,00	0,00	0,00	16,00
Gel energético con cafeína	Enervit	25	1 serving	71,20	-1,72	17,80	-1,69	0,00	0,00	0,00	0,00	30,00
Energy gel 100 BOX	Maurten	40	1 serving	100,00	-0,06	25,00	0,00	0,00	0,00	0,00	0,00	34,00
Energía Gel 44g Lemon-Fresh [40mg cafeína]	Nutrixxion	44	1 serving	118,95	1,03	29,60	1,08	0,14	0,50	0,00	0,00	36,00
Energía Gel 44g Cola Lemon [40mg cafeína]	Nutrixxion	44	1 serving	118,95	1,03	29,60	1,08	0,14	0,50	0,00	0,00	36,00
Energía Gel 44g XX Force Green Apple [80mg cafeína]	Nutrixxion	44	1 serving	117,14	0,93	28,51	0,83	0,77	2,80	0,00	0,00	36,00
Energía Gel 44g XX Force Original [80mg cafeína]	Nutrixxion	44	1 serving	117,14	0,93	28,51	0,83	0,77	2,80	0,00	0,00	36,00
Energía Gel 44g Lakritz [40mg cafeína]	Nutrixxion	44	1 serving	117,43	0,95	29,22	0,99	0,14	0,50	0,00	0,00	36,00
Energía Gel 44g Waldmeister	Nutrixxion	44	1 serving	118,95	1,03	29,60	1,08	0,14	0,50	0,00	0,00	36,00
Energía Gel 44g Orange [40mg cafeína]	Nutrixxion	44	1 serving	118,95	1,03	29,60	1,08	0,14	0,50	0,00	0,00	36,00
Energía Gel 44g Banana	Nutrixxion	44	1 serving	118,95	1,03	29,60	1,08	0,14	0,50	0,00	0,00	36,00
Energía Gel 44g Citrus	Nutrixxion	44	1 serving	92,40	-0,50	23,10	-0,45	0,00	0,00	0,00	0,00	0,00
Energía Gel 44g Vanilla-Strawberry	Nutrixxion	44	1 serving	92,40	-0,50	23,10	-0,45	0,00	0,00	0,00	0,00	0,00
Energie Gel 44g Strawberry	Nutrixxion	44	1 serving	102,00	0,06	25,50	0,12	0,00	0,00	0,00	0,00	0,00
Gel energético	Enervit	25	1 serving	98,40	-0,15	24,60	-0,09	0,00	0,00	0,00	0,00	0,00
Gel energy elite	Myprotein	50	1 serving	113,44	0,72	28,36	0,79	0,00	0,00	0,00	0,00	0,00

Energy boost gel + caffeine cola	Victory endurance	42	1 serving	121,60	1,19	30,40	1,27	0,00	0,00	0,00	0,00	0,00
Energy boost gel + caffeine red energy	Victory endurance	42	1 serving	96,00	-0,29	24,00	-0,24	0,00	0,00	0,00	0,00	0,00
				**100-200 mg sodium**								
Energy up gel lemon	Victory endurance	40	1 serving	104,00	0,17	26,00	0,24	0,00	0,00	0,00	0,00	100,00
Energy up gel naranja	Victory endurance	40	1 serving	104,00	0,17	26,00	0,24	0,00	0,00	0,00	0,00	100,00
Energy up gel sandía	Victory endurance	40	1 serving	104,00	0,17	26,00	0,24	0,00	0,00	0,00	0,00	100,00
Energy up! + caffeine mojito	Victory endurance	40	1 serving	96,00	-0,29	24,00	-0,24	0,00	0,00	0,00	0,00	100,00
Energy up! + caffeine cola	Victory endurance	40	1 serving	96,00	-0,29	24,00	-0,24	0,00	0,00	0,00	0,00	100,00
Energy up! + caffeine tropical	Victory endurance	40	1 serving	96,00	-0,29	24,00	-0,24	0,00	0,00	0,00	0,00	100,00
Energy Gel sabor cola con cafeína	Crown Sport Nutrition	40	1 serving	104,48	0,20	25,40	0,09	0,72	2,60	0,00	0,00	110,00
Energy Gel sabor Frutos Rojos con cafeína	Crown Sport Nutrition	40	1 serving	105,28	0,25	25,60	0,14	0,72	2,60	0,00	0,00	110,00
Energy Gel sabor limón	Crown Sport Nutrition	40	1 serving	106,48	0,32	25,90	0,21	0,72	2,60	0,00	0,00	112,00
Energy Gel sabor Naranja	Crown Sport Nutrition	40	1 serving	105,28	0,25	25,60	0,14	0,72	2,60	0,00	0,00	112,00
Bio energy gel - sabor frutas del bosque	226ers	25	1 serving	73,18	-1,60	18,08	-1,63	0,05	0,20	0,08	1,50	154,50
Bio energy gel - sabor café	226ers	25	1 serving	73,05	-1,61	18,13	-1,62	0,03	0,10	0,05	1,00	154,75
BIO ENERGY GEL - SABOR piña & COCO	226ERS	25	1 serving	73,75	-1,57	18,28	-1,58	0,05	0,20	0,05	1,00	154,75
Bio energy gel - sabor limon	226ers	25	1 serving	72,88	-1,62	18,00	-1,65	0,05	0,20	0,08	1,50	154,75
Bio energy gel - sabor fresa y plátano	226ers	25	1 serving	73,85	-1,56	18,30	-1,58	0,05	0,20	0,05	1,00	155,00
Bio energy gel - sabor cola	226ers	25	1 serving	73,45	-1,59	18,23	-1,59	0,03	0,10	0,05	1,00	157,00

Bio energy gel - sabor melon	226ers	25	1 serving	73,55	-1,58	18,25	-1,59	0,03	0,10	0,05	1,00	157,00
Bio energy gel - sabor mango	226ers	25	1 serving	73,75	-1,57	18,30	-1,58	0,03	0,10	0,05	1,00	157,35
GEL ORAL sabor limón	Infisport	50	1 serving	130,00	1,67	31,70	1,58	0,80	2,90	0,00	0,00	160,00
GEL ORAL sabor naranja	Infisport	50	1 serving	120,40	1,12	29,30	1,01	0,80	2,90	0,00	0,00	170,00
ND3 CROSS UP sabor frutos del bosque	Infisport	50	1 serving	124,36	1,35	30,20	1,22	0,89	3,20	0,00	0,00	180,00
ND3 CROSS UP sabor limón	Infisport	50	1 serving	121,52	1,18	29,65	1,09	0,73	2,60	0,00	0,00	185,00
GEL ORAL sabor cola	Infisport	50	1 serving	123,16	1,28	29,85	1,14	0,94	3,40	0,00	0,00	190,00
Gel energy	Recuperat-ion	32	1 serving	72,00	-1,67	18,00	-1,65	0,00	0,00	0,00	0,00	200,00
				**> 200 mg sodium**								
Isotonic energy gel	Etixx	40	1 serving	96,00	-0,29	24,00	-0,24	0,00	0,00	0,00	0,00	220,00
Bio energy gel - sabor regaliz	226ers	25	1 serving	73,05	-1,61	18,13	-1,62	0,03	0,10	0,05	1,00	241,00
Bio energy gel - sabor caramel	226ers	25	1 serving	74,65	-1,52	18,28	-1,58	0,05	0,20	0,15	3,00	243,25
Bio energy gel - sabor fresa y plátano	226ers	40	1 serving	118,10	0,99	29,30	1,01	0,00	0,00	0,10	2,00	248,00
Bio energy gel - sabor cola	226ers	40	1 serving	117,70	0,96	29,20	0,99	0,00	0,00	0,10	2,00	251,00
Bio energy gel - sabor melon	226ers	40	1 serving	117,70	0,96	29,20	0,99	0,00	0,00	0,10	2,00	251,00
Bio energy gel - sabor caramelo	226ers	40	1 serving	119,90	1,09	29,20	0,99	0,10	0,40	0,30	6,00	389,40
Dextro drink	Glucoup! - dextro energy	50	1 serving	96,00	-0,29	24,00	-0,24	0,00	0,00	0,00	0,00	
Gel energy & activation mixed berries	Keepgoing	32	1 serving	83,17	-1,03	20,50	-1,06	0,22	0,80	0,03	0,64	
Gel energy & endurance citrus	Keepgoing	32	1 serving	84,48	-0,95	20,80	-0,99	0,32	1,10	0,00	0,00	
Gel energy & recovery mandarine	Keepgoing	32	1 serving	84,17	-0,97	20,80	-0,99	0,22	0,80	0,01	0,20	
Longovit Gel Fresa y Plátano	Nutrinovex	60	1 serving	66,30	-2,00	15,80	-2,16	0,77	2,80	0,00	0,04	0,00
Longovit gel manzana	Nutrinovex	60	1 serving	82,4	-1,07	20	-1,18	0,6	2,1	0	0	0,144
			**Media**	**101,28**		**25,12**		**0,16**		**0,02**		
			**Deviation**	**17,35**		**4,25**		**0,28**		**0,05**		
			**Coefficient of Variation (CV)**	**17,14**		**16,93**		**175,73**		**249, 30**		

SPORTS BARS GROUP<br>Subgroup 1. <25 g carbohydrate			EXCHANGE VALUE	118	0,05	24,00	0,01	1	-0,24	2	0,08
**Produt name**	**Brand name**	**Net weight (g or ml)**	**Serving dosage**	**Energy (Kcal)**	**Z value**	**Carbohydrate (g)**	**Z value**	**Protein (g)**	**Z value**	**Fat (g)**	**Z value**
Longovit plátano	Nutrinovex	30	1 serving	124,71	0,13	18,06	-1,61	2,07	0,95	4,91	1,61
Longovit Tarta de queso	Nutrinovex	30	1 serving	119,99	-0,10	16,67	-1,99	2,10	0,97	4,99	1,65
Faster bar sabor frambuesa	Infisport	25	1 serving	78,10	-2,08	19,00	-1,36	0,30	-0,62	0,10	-1,05
Tropical	Geo	40	1 serving	111,30	-0,51	23,00	-0,27	1,00	0,00	1,70	-0,17
Manzana - canela	Geo	40	1 serving	117,00	-0,24	24,10	0,03	1,10	0,09	1,80	-0,11
Chocolate	Geo	40	1 serving	126,50	0,21	20,80	-0,87	2,50	1,33	3,70	0,94
Faster bar sabor naranja-limón	INFISPORT	25	1 serving	81,90	-1,90	20,00	-1,09	0,25	-0,66	0,10	-1,05
Energy sport bar chocolate	Etixx	40	1 serving	143,30	1,01	29,00	1,36	2,10	0,97	2,10	0,06
Energy sport bar orange	Etixx	40	1 serving	142,10	0,95	29,00	1,36	1,80	0,71	2,10	0,06
Energy fruit mixed berries	Keepgoing	40	1 serving	150,14	1,34	25,95	0,53	2,90	1,68	3,86	1,03
Energy fruit banana	Keepgoing	40	1 serving	137,77	0,75	24,84	0,23	2,65	1,46	3,09	0,60
Energy fruit orange	Keepgoing	40	1 serving	136,97	0,71	24,85	0,23	2,71	1,51	2,97	0,54
Glucobar fresa	Nutrinovex	35	1 serving	105,32	-0,79	26,18	0,59	0,07	-0,82	0,04	-1,09
Glucobar cola	Nutrinovex	35	1 serving	105,60	-0,78	26,32	0,63	0,00	-0,88	0,04	-1,09
Glucobar kiwi	Nutrinovex	35	1 serving	97,68	-1,16	24,34	0,09	0,00	-0,88	0,04	-1,09
Glucobar lima limón	Nutrinovex	35	1 serving	105,60	-0,78	26,32	0,63	0,00	-0,88	0,04	-1,09
Glucobar plátano	Nutrinovex	35	1 serving	105,12	-0,80	26,20	0,60	0,00	-0,88	0,04	-1,09
			**Media**	**117,00**		**23,80**		**1,27**		**1,86**	
			**Deviation**	**21,06**		**3,68**		**1,13**		**1,81**	

**Coefficient of Variation (CV)**	**18,00**		**15,45**		**89,12**		**97,21**	

**SPORTS BARS GROUP**<br>Subgroup 2. >30 g carbohydrate, 2 g of fat, and 2 g of protein.			**EXCHANGE VALUE**	**166**	**-0,04**	**35,00**	**0,08**	**2**	**-0,15**	**2**	**-0,15**
**Produt name**	**Brand name**	**Net weight (g or ml)**	**Serving dosage**	**Energy (Kcal)**	**Z value**	**Carbohydrate (g)**	**Z value**	**Protein (g)**	**Z value**	**Fat (g)**	**Z value**
Energy sport bar lemon	Etixx	40	1 serving	153,3	-0,4	30,0	-1,1	1,8	-0,1	2,9	0,5
Energy sport bar raspberry	Etixx	40	1 serving	145,2	-0,5	30,0	-1,1	1,8	-0,1	2,0	0,0
Evo bar - oat & prebiotics - multifrutas	226ers	60	1 serving	214,8	0,8	41,9	1,5	4,1	1,5	3,4	0,8
Enervit power sport competition – naranja (sin gluten)	Enervit	60	1 serving	210,1	0,7	46,2	2,5	2,3	0,2	1,8	-0,1
ND3 SOLID sabor citrico	INFISPORT	40	1 serving	127,3	-0,8	30,3	-1,0	0,4	-1,1	0,5	-0,9
ND3 SOLID sabor citrico cafeina	INFISPORT	40	1 serving	128,5	-0,8	30,6	-1,0	0,4	-1,1	0,5	-0,9
ND3 SOLID granada	INFISPORT	40	1 serving	130,5	-0,8	31,0	-0,9	0,5	-1,1	0,5	-0,9
ND3 SOLID granada cafeina	INFISPORT	40	1 serving	122,1	-0,9	30,1	-1,1	0,4	-1,1	0,0	-1,2
Fruit bar	Infisport	40	1 serving	136,1	-0,7	30,8	-0,9	1,2	-0,6	0,9	-0,7
Energia barrita fruit	Nutrixxion	55	1 serving	187,1	0,2	35,3	0,1	3,0	0,7	3,8	1,1
Energía barrita banana 55g	Nutrixxion	55	1 serving	188,7	0,3	35,6	0,1	2,9	0,7	3,9	1,1
Energía barrita salty nut 55g	Nutrixxion	55	1 serving	188,0	0,3	33,9	-0,3	3,9	1,3	4,1	1,3
Endurance fuel bar con bcaa's - plátano y jengibre	226ers	60	1 serving	165,8	-0,1	37,3	0,5	4,0	1,4	0,1	-1,1
Endurance fuel bar con bcaa's - manzana y canela	226ers	60	1 serving	195,5	0,4	41,3	1,4	3,4	1,0	1,9	-0,1

Endurance fuel bar con bcaa's - chocolate	226ers	60	1 serving	201,5	0,5	35,3	0,1	5,0	2,1	4,5	1,5
Endurance fuel bar con choco bits - limon	226ers	60	1 serving	197,3	0,4	36,3	0,3	3,3	0,9	4,3	1,4
Endurance fuel bar con choco bits - cafe y cacao	226ers	60	1 serving	191,0	0,3	37,8	0,6	2,9	0,7	3,1	0,7
Endurance fuel bar con white choco bits - fresa	226ers	60	1 serving	153,3	-0,4	30,0	-1,1	1,8	-0,1	2,9	0,5
Endurance fuel bar con chia - sabor italian	226ers	60	1 serving	145,2	-0,5	30,0	-1,1	1,8	-0,1	2,0	0,0
Evo bar - superfoods energy- arandanos, nuecs y maca	226ers	50	1 serving	214,8	0,8	41,9	1,5	4,1	1,5	3,4	0,8
Evo bar - superfoods energy - plátano, avellana y maca	226ers	50	1 serving	210,1	0,7	46,2	2,5	2,3	0,2	1,8	-0,1
Triforza bar strawberry	Keepgoing	40	1 serving	127,3	-0,8	30,3	-1,0	0,4	-1,1	0,5	-0,9
Triforza bar mandarine	Keepgoing	40	1 serving	128,5	-0,8	30,6	-1,0	0,4	-1,1	0,5	-0,9
Triforza bar albaricoque	Keepgoing	40	1 serving	130,5	-0,8	31,0	-0,9	0,5	-1,1	0,5	-0,9
			**Media**	**167,5**		**34,6**		**2,2**		**2,2**	
			**Desviación**	**33,3**		**4,5**		**1,4**		**1,7**	
			**Coefficient of Variation (CV)**	**19,9**		**13,0**		**64,7**		**76,3**	

**SPORTS BARS GROUP** Subgroup 3. >30 g carbohydrate, 6 g of fat, and 5 g of protein.			**EXCHANGE VALUE**	**206**	**0,10**	**33,0**	**0,0**	**5,0**	**0,2**	**6,0**	**0,0**
**Produt name**	**Brand name**	**Net weight (g or ml)**	**Serving dosage**	**Energy (Kcal)**	**Z value**	**Carbohydrate (g)**	**Z value**	**Protein (g)**	**Z value**	**Fat (g)**	**Z value**
Energy Bar sabor doble chocolate	Crown Sport Nutrition	60	1 serving	228,92	1,07	32,30	-0,16	5,76	0,33	8,52	1,68
Energy Bar sabor yogur	Crown Sport Nutrition	60	1 serving	227,12	0,99	32,90	-0,02	5,52	0,23	8,16	1,44
Energy Bar sabor banana con choco blanco	Crown Sport Nutrition	60	1 serving	228,18	1,04	35,10	0,48	5,34	0,15	7,38	0,92

Energy sport bar nougat	Etixx	40	1 serving	179,60	-1,23	29,00	-0,91	2,40	-1,13	6,00	0,00
Nature´s energy bar fresa	Victory endurance	60	1 serving	187,80	-0,85	29,00	-0,91	3,10	-0,83	6,60	0,40
Nature´s energy bar manzana	Victory endurance	60	1 serving	187,80	-0,85	29,00	-0,91	3,10	-0,83	6,60	0,40
Energía barrita fruit joghurt.	Nutrixxion	55	1 serving	212,80	0,32	34,49	0,34	2,75	-0,98	7,10	0,73
Energía barrita oat raisin	Nutrixxion	55	1 serving	212,69	0,31	35,30	0,53	3,10	-0,81	6,50	0,36
Barritas con proteínas – chocolate	Enervit	40	1 serving	161,20	-2,09	23,20	-2,23	7,20	0,96	4,40	-1,07
Evo bar - superfoods protein -arandanos, nuecs y maca	226ERS	50	1 serving	185,75	-0,95	32,05	-0,22	7,30	1,00	3,15	-1,90
Enervit power sport – manzana	Enervit	60	1 serving	221,16	0,71	37,20	0,95	10,80	2,52	3,24	-1,84
Energía Barrita Peanut Choco 55g	Nutrixxion	55	1 serving	196,41	-0,45	31,57	-0,33	4,79	-0,09	5,67	-0,22
Nutrixxion energía barrita cappuccino [40mg koffein]	Nutrixxion	55	1 serving	215,93	0,46	38,01	1,14	2,86	-0,93	5,83	-0,11
Energy bar	Infisport	40	1 serving	176,45	-1,38	29,20	-0,86	1,93	-1,33	5,77	-0,15
Endurance fuel bar con chia - sabor indian	226ERS	60	1 serving	197,34	-0,40	32,94	-0,01	4,38	-0,27	5,34	-0,44
Endurance fuel bar - patata y quiqos	226ERS	60	1 serving	219,90	0,65	38,70	1,30	3,72	-0,56	5,58	-0,28
Evo bar - oat & prebiotics - plátano y chocolate blanco	226ers	60	1 serving	228,00	1,03	40,92	1,80	4,20	-0,35	5,28	-0,48
			**Media**	**203,9**		**33,0**		**4,6**		**6,0**	
			**Deviation**	**21,4**		**4,4**		**2,3**		**1,5**	
			**Coefficient of Variation (CV)**	**10,5**		**13,4**		**49,0**		**24,8**	

**SPORTS CONFECTIONERY GROUP**			**EXCHANGE VALUE**	**20**	**0,02**	**5,00**	**0,28**	**0,00**	**-0,60**	**0,00**	**-0,70**
**Produt name**	**Brand name**	**Net weight (g or ml)**	**Serving dosage**	**Energy (Kcal)**	**Z value**	**Carbohydrate (g)**	**Z value**	**Protein (g)**	**Z value**	**Fat (g)**	**Z value**
Glucotabs	Glucoup! - gluco	4	1 serving	14,40	-0,97	3,60	-1,09	0,00	0,00	0,00	0,00

Dextrose cubes	Glucoup! - dextro energy	5,45	1 serving	25,30	0,92	5,20	0,16	0,00	0,00	0,50	3,13
Dixi	Glucoup! - instantina	4,68	4 serving	16,04	-0,69	3,92	-0,84	0,00	0,00	0,04	0,25
Dixi max	Glucoup! - instantina	5,2	2 serving	19,08	-0,16	4,68	-0,25	0,00	0,00	0,04	0,25
Clio	Glucoup! - instantina	4,96	2 serving	19,40	-0,10	4,40	-0,47	0,00	0,00	0,20	1,25
Vegan sport gummies - mango	226ers	6	1 serving	13,80	-1,08	3,34	-1,29	0,08	0,70	0,01	0,08
Vegan sport gummies - cereza y cola	226ERS	6	1 serving	14,94	-0,88	3,33	-1,29	0,38	3,15	0,01	0,08
Energy boost gummies	Victory endurance	8	1 serving	27,90	1,37	6,64	1,27	0,11	0,92	0,10	0,63
Energy boost gummies caffeine	Victory endurance	8	1 serving	27,86	1,37	6,64	1,27	0,10	0,83	0,10	0,63
			**Media**	**19,86**		**4,64**		**0,07**		**0,11**	
			**Deviation**	**5,75**		**1,29**		**0,12**		**0,16**	
			**Coefficient of Variation (CV)**	**28,95**		**27,88**		**165,30**		**142,26**	

**PROTEIN POWDERS GROUP**<br>Subgroup 1. Between 20 and <25 g protein.			**EXCHANGE VALUE**	**109**	**0,03**	**2,00**	**-0,10**	**23,00**	**0,31**	**1**	**-0,1606889**
**Produt name**	**Brand name**	**Net weight (g or ml)**	**Serving dosage**	**Energy (Kcal)**	**Z value**	**Carbohydrate (g)**	**Z value**	**Protein (g)**	**Z value**	**Fat (g)**	**Z value**
Day & night casein chocolate	Weider	25	1 serving	89,30	-2,02	1,20	-0,85	20,00	-2,59	0,50	-0,75
Isolate protein drink - chocolate	226ers	25	2 serving	90,40	-1,91	2,35	0,37	20,25	-2,37	0,00	-1,49
Sequential Protein chocolate	Crown Sport Nutrition	27	1 serving	98,07	-1,12	1,34	-0,70	20,41	-2,23	1,23	0,34
Impact Whey protein	Myprotein	25	1 serving	102,88	-0,63	1,00	-1,06	20,50	-2,16	1,88	1,31
The whey +	Myprotein	32	1 serving	91,33	-1,81	1,60	-0,43	20,50	-2,16	0,33	-1,01
Vegan protein - chocolate	226ers	30	2 serving	102,60	-0,66	3,60	1,70	20,70	-1,98	0,60	-0,60
Lean Protein chocolate	Weider	30	1 serving	100,10	-0,91	2,00	0,00	21,00	-1,72	0,90	-0,15
Enervit proteina 100% whey	Enervit	30	1 serving	119,82	1,11	4,50	2,66	21,00	-1,72	1,98	1,46

Vegan protein - red fruits	226ers	30	2 serving	104,43	-0,47	4,20	2,34	21,30	-1,47	0,27	-1,09
Sequential Protein fresa	Crown Sport Nutrition	27	1 serving	101,34	-0,79	1,63	-0,39	21,50	-1,29	0,98	-0,03
Iso whey hydrolyzed chocolate	Crown sport nutrition	27	1 serving	97,50	-1,18	0,80	-1,28	22,00	-0,86	0,70	-0,45
Day & night casein frutas del bosque	Weider	25	1 serving	93,85	-1,56	0,90	-1,17	22,00	-0,86	0,25	-1,12
Day & night casein vainilla	Weider	25	1 serving	93,45	-1,60	0,80	-1,28	22,00	-0,86	0,25	-1,12
Super nitro whey chocolate praliné	Victory endurance	30	1 serving	116,30	0,75	2,80	0,85	22,00	-0,86	1,90	1,34
ISO WHEY PROTEIN Yogur frutos del bosque	Keepgoing	30	2 serving	101,40	-0,78	2,00	0,00	22,00	-0,86	0,60	-0,60
Iso whey protei chocolate	Keepgoing	30	2 serving	112,00	0,31	1,50	-0,53	22,00	-0,86	2,00	1,49
Impact Whey isolate	Myprotein	25	1 serving	93,18	-1,62	0,63	-1,46	22,50	-0,43	0,08	-1,38
Caseína de acción lenta	Myprotein	30	1 serving	100,05	-0,92	1,20	-0,85	22,80	-0,17	0,45	-0,82
Beef & whey	Crown sport nutrition	27	1 serving	104,20	-0,49	0,80	-1,28	23,00	0,00	1,00	0,00
Iso whey hydrolyzed fresa	Crown sport nutrition	27	1 serving	100,00	-0,92	1,10	-0,96	23,00	0,00	0,40	-0,90
Gold whey banana	Weider	30	1 serving	117,40	0,86	2,30	0,32	23,00	0,00	1,80	1,19
Gold whey chocolate	Weider	30	1 serving	119,90	1,12	2,70	0,74	23,00	0,00	1,90	1,34
Gold whey coco-cookie	Weider	30	1 serving	122,90	1,43	3,00	1,06	23,00	0,00	2,10	1,64
Gold whey fresa	Weider	30	1 serving	117,30	0,85	2,50	0,53	23,00	0,00	1,70	1,04
Gold whey mango-maracuya	Weider	30	1 serving	116,50	0,77	2,30	0,32	23,00	0,00	1,70	1,04
Gold whey stracciatella	Weider	30	1 serving	122,90	1,43	3,00	1,06	23,00	0,00	2,10	1,64
Gold whey vainilla	Weider	30	1 serving	117,00	0,82	2,20	0,21	23,00	0,00	1,80	1,19
Lean Protein vainilla	Weider	30	1 serving	107,20	-0,18	2,00	0,00	23,00	0,00	0,80	-0,30
Pro whey complex fresa	Victory endurance	30	2 serving	113,30	0,44	3,30	1,38	23,00	0,00	0,90	-0,15
Pro whey complex vainilla	Victory endurance	30	2 serving	113,30	0,44	3,30	1,38	23,00	0,00	0,90	-0,15
Protein whey complex	Weider	30	1 serving	116,50	0,77	3,20	1,28	23,00	0,00	1,30	0,45

Vegan protein chocolate	Weider	30	1 serving	103,80	-0,53	1,60	-0,43	23,00	0,00	0,60	-0,60
Super nitro whey fresa-banana	Victory endurance	30	1 serving	121,70	1,30	3,60	1,70	23,00	0,00	1,70	1,04
Super nitro whey crema-vainilla	Victory endurance	30	1 serving	120,90	1,22	3,40	1,49	23,00	0,00	1,70	1,04
Super nitro whey cookies&cream	Victory endurance	30	1 serving	121,00	1,23	3,20	1,28	23,00	0,00	1,80	1,19
Premium whey chocolate-turrón	Weider	30	1 serving	116,00	0,72	2,40	0,43	23,00	0,00	1,60	0,90
Iso whey protei capuchino	Keepgoing	30	2 serving	115,90	0,71	1,70	-0,32	23,00	0,00	1,90	1,34
Iso whey protei vainilla	Keepgoing	30	2 serving	97,60	-1,17	1,40	-0,64	23,00	0,00		-1,49
Proteina Bebida Whey Isolate 100 Banana-Strawberry 450g	Nutrixxion	30	2 serving	107,14	-0,19	2,34	0,36	23,76	0,65	0,31	-1,03
100% all beef	Crown sport nutrition	30	3 seving	117,60	0,88	0,90	-1,17	23,55	0,47	2,20	1,79
Proteína de suero hidrolizada	Myprotein	30	1 serving	112,53	0,36	0,96	-1,11	24,00	0,86	1,41	0,61
Gold whey frambuesa-yogur	Weider	30	1 serving	121,70	1,30	2,60	0,64	24,00	0,86	1,70	1,04
Pro whey complex chocolate	Victory endurance	30	2 serving	110,46	0,15	1,68	-0,34	24,00	0,86	0,86	-0,21
Protein 80 plus chocolate	Weider	30	2 serving	108,90	-0,01	2,10	0,11	24,00	0,86	0,50	-0,75
Protein 80 plus fresa	Weider	30	2 serving	108,90	-0,01	2,10	0,11	24,00	0,86	0,50	-0,75
Protein 80 plus vainilla	Weider	30	2 serving	109,70	0,07	2,30	0,32	24,00	0,86	0,50	-0,75
Vegan protein vainilla	Weider	30	1 serving	105,60	-0,35	1,50	-0,53	24,00	0,86	0,40	-0,90
Premium whey fresa-vainilla	Weider	30	1 serving	115,70	0,69	2,00	0,00	24,00	0,86	1,30	0,45
Premium whey vainilla-caramelo	Weider	30	1 serving	118,40	0,97	2,00	0,00	24,00	0,86	1,60	0,90
High protein shake vainilla	Etixx	30	1 serving	106,00	-0,31	1,30	-0,74	24,30	1,12	0,40	-0,90
			**Media**	**108,72**		**2,10**		**22,64**		**1,11**	
			**Deviation**	**9,74**		**0,94**		**1,16**		**0,67**	
			**Coefficient of Variation (CV)**	**8,95**		**45,03**		**5,11**		**60,34**	

PROTEIN POWDERS GROUP<br>Subgroup 2. Between ≥25 and 30 g protein			EXCHANGE VALUE	119	0,28	2,00	0,36	26,00	0,18	0,5	-0,26
**Produt name**	**Brand name**	**Net weight (g or ml)**	**Serving dosage**	**Energy (Kcal)**	**Z value**	**Carbohydrate (g)**	**Z value**	**Protein (g)**	**Z value**	**Fat (g)**	**Z value**
Iso pro-t sabor chocolate	Infisport	30	3 serving	111,45	-0,59	2,30	0,29	25,00	-1,00	0,25	-0,33
Iso pro-t sabor fresa	Infisport	30	3 serving	105,20	-1,08	1,30	-0,67	25,00	-1,00	0,00	-0,66
Iso whey leucina+ sabor chocolate	Infisport	34	3 serving	129,60	0,83	2,00	0,00	25,00	-1,00	2,40	2,50
Iso whey leucina+ sabor fresa	Infisport	33	3 serving	109,20	-0,76	2,30	0,29	25,00	-1,00	0,00	-0,66
Iso whey leucina+ sabor mango	Infisport	33	3 serving	124,40	0,42	1,60	-0,38	25,00	-1,00	2,00	1,97
Isolate whey 100 cfm sabor chocolate fondant	Weider	30	1 serving	110,80	-0,64	0,90	-1,05	25,00	-1,00	0,80	0,39
Isolate whey 100 cfm sabor strawberry ice cream	Weider	30	1 serving	109,70	-0,73	1,30	-0,67	25,00	-1,00	0,50	0,00
Protein 80 plus frutos del bosque	Weider	30	2 serving	112,90	-0,48	2,10	0,10	25,00	-1,00	0,50	0,00
Protein 80 plus plátano	Weider	30	2 serving	113,70	-0,41	2,30	0,29	25,00	-1,00	0,50	0,00
Protein drink whey isolate 100 450g raspberry-blackberry	Nutrixxion	30	2 serving	108,42	-0,83	1,32	-0,65	25,12	-0,88	0,30	-0,27
Isolate whey 100 cfm sabor vanilla cream	Weider	30	1 serving	110,30	-0,68	0,90	-1,05	26,00	0,00	0,30	-0,26
Isolate whey 100 cfm sabor cookies & cream	Weider	30	1 serving	114,10	-0,38	1,40	-0,57	26,00	0,00	0,50	0,00
Neo isolate whey chocolate	Victory endurance	30	1 serving	111,60	-0,58	1,00	-0,95	26,00	0,00	0,40	-0,13
Neo isolate whey vainilla	Victory endurance	30	1 serving	110,70	-0,65	1,00	-0,95	26,00	0,00	0,30	-0,26
Proteina bebida whey isolate 100 schoko-nocciola 450g	Nutrixxion	30	2 serving	111,94	-0,55	1,02	-0,93	26,14	0,14	0,36	-0,18
Soy protein isolate	Myprotein	30	1,25 serving	114,90	-0,32	0,30	-1,62	26,40	0,40	0,90	0,53
Proteina bebida whey isolate 100 vanilla 450g	Nutrixxion	30	2 serving	111,65	-0,57	0,58	-1,36	26,72	0,72	0,27	-0,30
Neo isolate whey fresa	Victory endurance	30	1 serving	114,70	-0,34	1,00	-0,95	27,00	1,00	0,30	-0,26
Protein secuencial sabor chocolate	Infisport	40	3 serving	146,30	2,13	2,50	0,48	28,00	2,00	2,70	2,89

K-weeks immune - chocolate	226ers	40	2 serving	138,56	1,53	5,20	3,05	28,00	2,00	0,64	0,18
			**Media**	**116,01**		**1,62**		**25,82**		**0,70**	
			**Deviation**	**10,58**		**1,05**		**1,00**		**0,76**	
			**Coefficient of Variation (CV)**	**9,12**		**65,19**		**3,86**		**109,29**	

**PROTEIN BARS GROUP**<br>Subgroup 1. <15 g protein per serving.	**EXCHANGE VALUE**			**161**	**0,10**	**18,00**	**0,02**	**11,00**	**0,09**	**5,00**	**0,11**
**Produt name**	**Brand name**	**Net weight (g or ml)**	**Serving dosage**	**Energy (Kcal)**	**Z value**	**Carbohydrate (g)**	**Z value**	**Protein (g)**	**Z value**	**Fat (g)**	**Z value**
Vegan protein bar frutos rojos	Victory endurance	35	1 serving	136,20	-0,97	16,00	-0,46	7,70	-2,10	4,60	-0,23
Vegan protein bar cacahuete salado	Victory endurance	35	1 serving	154,90	-0,24	13,00	-1,14	8,40	-1,66	7,70	1,53
Barritas proteicas avellana	Weider	35	1 serving	146,90	-0,55	16,00	-0,46	8,80	-1,40	5,30	0,17
Barritas proteicas chocolate blanco	Weider	35	1 serving	142,00	-0,74	17,00	-0,23	9,50	-0,96	4,00	-0,57
Chocolate blanco	Weider	35	1 serving	142,00	-0,74	17,00	-0,23	9,50	-0,96	4,00	-0,57
Classic bar banana	Weider	35	1 serving	142,90	-0,71	17,00	-0,23	9,50	-0,96	4,10	-0,51
Cacao y reishi	Paleobull	50	1 serving	201,04	1,56	22,71	1,08	10,01	-0,63	7,80	1,59
Protein bar sabor fresa-chocolate	Infisport	40	1 serving	156,32	-0,18	17,92	-0,02	10,72	-0,18	4,64	-0,20
Nutrixxion proteina (bajo en azúcar) barrita cheesecake 35g	Nutrixxion	35	1 serving	124,15	-1,44	10,75	-1,66	10,92	-0,05	4,17	-0,47
Nutrixxion proteina (bajo en azúcar) barrita choco caramel	Nutrixxion	35	1 serving	122,40	-1,51	10,68	-1,67	11,03	0,02	3,96	-0,59
Nutrixxion proteina (bajo en azúcar) barrita toffee	Nutrixxion	35	1 serving	122,40	-1,51	10,68	-1,67	11,03	0,02	3,96	-0,59
Vegan protein bar - cacao	226ers	40	1 serving	148,16	-0,50	16,28	-0,39	11,04	0,03	4,32	-0,39
Protein "low sugar" riegel 35g coconut	Nutrixxion	35	1 serving	123,52	-1,46	10,61	-1,69	11,06	0,04	4,10	-0,51
Barritas con proteínas – yogur y vainilla	Enervit	40	1 serving	152,36	-0,34	19,60	0,37	11,20	0,13	3,24	-1,00
Protein bar sabor chocolate-chocolate	Infisport	40	1 serving	174,64	0,53	18,40	0,09	11,40	0,25	6,16	0,66

Vegan protein bar - frambuesa	226ers	40	1 serving	148,00	-0,51	19,04	0,24	11,48	0,31	2,88	-1,20
Recovery bar mani	Etixx	40	1 serving	160,00	-0,04	19,00	0,23	12,00	0,64	4,00	-0,57
Protein rocky road bar	Myprotein	50	1 serving	230,00	2,70	23,00	1,14	12,00	0,64	10,00	2,84
Enervit power sport barrita proteina – chocolate y crema	Enervit	45	1 serving	178,97	0,70	18,41	0,09	12,06	0,68	6,35	0,76
Naranja y chía	Paleobull	50	1 serving	184,94	0,94	21,88	0,88	12,39	0,89	5,32	0,18
Vegan carb crusher	Myprotein	50	1 serving	169,00	0,31	14,00	-0,91	12,50	0,96	7,00	1,14
Recovery plus choco & coconut	Keepgoing	40	1 serving	177,00	0,63	22,40	1,00	12,40	0,89	4,20	-0,45
Recovery bar caramelo	Etixx	40	1 serving	165,00	0,16	17,00	-0,23	13,00	1,27	5,00	0,00
Flapjack ligera	Myprotein	50	1 serving	152,85	-0,32	24,50	1,48	10,00	-0,64	1,65	-1,90
Melocotón	Paleobull	50	1 serving	184,63	0,92	24,01	1,37	12,55	0,99	4,27	-0,42
Enervit power sport – crunchy cookie	Enervit	40	1 serving	150,88	-0,40	24,00	1,37	7,60	-2,17	2,72	-1,30
Café y guaraná	Paleobull	50	1 serving	186,12	0,98	23,18	1,18	13,45	1,56	4,40	-0,34
			**Media**	**158,42**		**17,93**		**10,86**		**4,81**	
			**Deviation**	**25,60**		**4,38**		**1,57**		**1,76**	
			**Coefficient of Variation (CV)**	**16,16**		**24,42**		**14,50**		**36,55**	

**PROTEIN BARS GROUP** Subgroup 2. >20 g protein per serving.	**EXCHANGE VALUE**			**192,00**	**0,00**	**14,00**	**0,10**	**23,00**	**0,10**	**5,00**	**-0,15**
**Produt name**	**Brand name**	**Net weight (g or ml)**	**Serving dosage**	**Energy (Kcal)**	**Z value**	**Carbohydrate (g)**	**Z value**	**Protein (g)**	**Z value**	**Fat (g)**	**Z value**
40% low carb high protein bar cacahuete-caramelo	Weider	50	1 serving	163,10	-0,50	11,10	-0,75	20,00	-0,97	4,30	-0,57
40% low carb high protein bar stracciatela	Weider	50	1 serving	164,10	-0,48	10,00	-1,03	20,00	-0,97	4,90	-0,08

40% low carb high protein bar frutos rojos	Weider	50	1 serving	164,30	-0,48	11,40	-0,67	20,00	-0,97	4,30	-0,57
High protein bar coconut	Etixx	50	1 serving	186,00	-0,10	13,00	-0,26	20,00	-0,97	6,00	0,81
High protein bar vainilla	Etixx	50	1 serving	186,00	-0,10	13,00	-0,26	20,00	-0,97	6,00	0,81
The carb crusher (caramelo con nueces)	Myprotein	60	1 serving	207,60	0,27	12,00	-0,52	21,00	-0,65	8,40	2,76
Neo bar - chocolate	226ers	50	1 serving	192,85	0,01	12,35	-0,43	24,95	0,63	4,85	-0,12
Neo bar - banana . Flavour y chocolate	226ers	50	1 serving	193,90	0,03	12,45	-0,40	25,00	0,65	4,90	-0,08
Barrita de rendimiento todo-en-uno	Myprotein	70	1 serving	260,47	1,19	23,10	2,35	28,00	1,62	6,23	1,00
Barrita proteica elite (caramelo y avellana)	Myprotein	70	1 serving	256,20	1,11	22,40	2,16	25,90	0,94	7,00	1,63
52% protein bar stracciatella	Weider	50	1 serving	196,80	0,08	14,20	0,05	26,00	0,97	4,00	-0,81
52% protein bar cacahuete-caramelo	Weider	50	1 serving	203,60	0,20	15,00	0,26	26,00	0,97	4,40	-0,49
52% protein bar chocolate	Weider	50	1 serving	201,80	0,17	15,00	0,26	26,00	0,97	4,20	-0,65
40% low carb high protein bar cacahuete-caramelo	Weider	50	1 serving	163,10	-0,50	11,10	-0,75	20,00	-0,97	4,30	-0,57
40% low carb high protein bar stracciatela	Weider	50	1 serving	164,10	-0,48	10,00	-1,03	20,00	-0,97	4,90	-0,08
40% low carb high protein bar frutos rojos	Weider	50	1 serving	164,30	-0,48	11,40	-0,67	20,00	-0,97	4,30	-0,57
			**Media**	**191,76**		**13,59**		**22,68**		**5,19**	
			**Deviation**	**57,69**		**3,88**		**3,08**		**1,23**	
			**Coefficient of Variation (CV)**	**0,00**		**28,57**		**13,59**		**23,63**	

**LIQUID MEALS GROUP**	**EXCHANGE VALUE**	**176**	**0,00**	**18**	**-0,19**	**21**	**0,26**	**2**	**-0,42**

Subgroup 1. Ratio of 1 Carbohydrate/1 protein.											
**Produt name**	**Brand name**	**Net weight (g or ml)**	**Serving dosage**	**Energy (Kcal)**	**Z value**	**Carbohydrate (g)**	**Z value**	**Protein (g)**	**Z value**	**Fat (g)**	**Z value**
Top 50/50 recovery 1:1 leucina+ sabor chocolate	Infisport	40	2 serving	145,38	-2,11	15,76	-1,52	17,84	-2,43	1,22	-1,32
Recovery drink - fresa	226ers	50	4 serving	184,50	0,59	19,50	1,02	21,00	0,00	2,50	0,85
Recovery drink - choco	226ers	50	4 serving	179,20	0,22	18,00	0,00	20,50	-0,38	2,80	1,36
Recovery drink- sandía	226ers	50	4 serving	184,50	0,59	19,00	0,68	21,50	0,38	2,50	0,85
Recovery drink - vainilla	226ers	50	4 serving	185,40	0,65	18,50	0,34	22,00	0,77	2,60	1,02
Recovery drink yogur de limon	226ers	50	4 serving	181,60	0,39	20,00	1,36	20,00	-0,77	2,40	0,68
Recovery drink - vanilla y cafe	226ers	50	4 serving	184,95	0,62	19,00	0,68	21,50	0,38	2,55	0,93
Full training complex shake soy-chocolate	Etixx	50	4 serving	162,60	-0,92	16,50	-1,02	21,00	0,00	1,40	-1,02
			**Media**	**176,02**		**18,28**		**20,67**		**2,25**	
			**Deviation**	**14,50**		**1,47**		**1,30**		**0,59**	
			**Coefficient of Variation (CV)**	**8,24**		**8,04**		**6,30**		**26,31**	

**LIQUID MEALS GROUP** Subgroup 2. Ratio of 2-4 Carbohydrate/1 protein.			**EXCHANGE VALUE**	**154,5**	**0,00**	**28,00**	**-0,03**	**10**	**0,15**	**0,5**	**0,38**
**Produt name**	**Brand name**	**Net weight (g or ml)**	**Serving dosage**	**Energy (Kcal)**	**Z value**	**Carbohydrate (g)**	**Z value**	**Protein (g)**	**Z value**	**Fat (g)**	**Z value**
3:1 pro recovery	Crown sport nutrition	59	1 serving	148,10	-0,35	25,30	-0,70	10,60	0,34	0,50	0,00
Roubaix	Geo	40	3 serving	128,00	-1,46	22,00	-1,56	10,00	0,00	0,00	-1,28
Complex 4:1 recovery sabor fresa	Infisport	40	3 serving	151,85	-0,15	30,00	0,52	7,40	-1,49	0,25	-0,64
Complex 4:1 recovery sabor chocolate	Infisport	40	3 serving	145,70	-0,49	27,00	-0,26	7,40	-1,49	0,90	1,03

Complex 4:1 recovery salts	Infisport	40	3 serving	143,45	-0,61	28,00	0,00	7,30	-1,55	0,25	-0,64
Recovery 3:1	Recuperat-ion	50	2 serving	181,60	1,50	33,60	1,45	11,80	1,03	0,00	-1,28
Recuperador vegano	Myprotein	52	1 serving	183,04	1,58	31,20	0,83	14,56	2,62	0,00	-1,28
Enervit r1 sport	Enervit	45	3 serving	148,96	-0,31	28,17	0,04	9,07	-0,53	0,00	-1,28
Premium amino intra workout fresh orange	Weider	40	2 serving	137,20	-0,96	24,00	-1,04	10,30	0,17	0,00	-1,28
Premium amino intra workout tropical	Weider	40	2 serving	137,20	-0,96	24,00	-1,04	10,30	0,17	0,00	-1,28
Mass gainer chocolate	Weider	50	1 serving	177,45	1,27	32,00	1,04	10,00	0,00	1,05	1,41
Mass gainer fresa	Weider	50	1 serving	177,45	1,27	32,00	1,04	10,00	0,00	1,05	1,41
Recovery shake chocolate	Etixx	50	3 serving	182,41	1,54	35,90	2,05	8,60	-0,80	0,49	-0,03
Quick recovery sandia	Keepgoing	40	2 serving	147,19	-0,40	27,07	-0,24	9,30	-0,40	0,19	-0,79
Quick recovery chocolate	Keepgoing	40	2 serving	143,38	-0,61	23,68	-1,12	10,14	0,08	0,90	1,03
Quick recovery vainilla-canela	Keepgoing	40	2 serving	147,44	-0,39	27,01	-0,26	9,40	-0,34	0,20	-0,77
Quick recovery piña	Keepgoing	40	2 serving	147,47	-0,39	27,00	-0,26	9,44	-0,32	0,19	-0,79
			**Media**	**154,58**		**28,11**		**9,74**		**0,35**	
			**Deviation**	**18,09**		**3,85**		**1,74**		**0,39**	
			**Coefficient of Variation (CV)**	**11,70**		**13,71**		**17,83**		**111,56**	
